# Whole-Genome Sequencing Among Kazakhstani Children with Early-Onset Epilepsy Revealed New Gene Variants and Phenotypic Variability

**DOI:** 10.1007/s12035-023-03346-3

**Published:** 2023-04-24

**Authors:** Mirgul Bayanova, Aidos K. Bolatov, Assiya Bazenova, Lyazzat Nazarova, Alissa Nauryzbayeva, Naanlep Matthew Tanko, Saule Rakhimova, Nazerke Satvaldina, Diana Samatkyzy, Ulan Kozhamkulov, Ulykbek Kairov, Ainur Akilzhanova, Dos Sarbassov

**Affiliations:** 1 University Medical Center CF, Kerey-Zhanibek Handar St. 5/1, Z05P3Y4, Astana, Kazakhstan; 2 Astana Medical University, Beybitshilik St. 49A, Z10K9D9, Astana, Kazakhstan; 3 Department of Biomedical Sciences, School of Medicine, Nazarbayev University, Astana, Kazakhstan 010000; 4 Center for Life Sciences, National Laboratory Astana, Nazarbayev University, Kabanbay batyr Ave 53, Astana, Kazakhstan 010000; 5 School of Sciences and Humanities, Nazarbayev University, Kabanbay batyr Ave 53, Astana, Kazakhstan 010000

**Keywords:** Epilepsy, Genetics, Whole-genome sequencing, Early onset epilepsy, Next-generation sequencing

## Abstract

In Kazakhstan, there is insufficient data on genetic epilepsy, which has its own clinical and management implications. Thus, this study aimed to use whole genome sequencing to identify and evaluate genetic variants and genetic structure of early onset epilepsy in the Kazakhstani pediatric population. In this study, for the first time in Kazakhstan, whole genome sequencing was carried out among epilepsy diagnosed children. The study involved 20 pediatric patients with early onset epilepsy and no established cause of the disease during the July–December, 2021. The average age at enrolment was 34.5 months, with a mean age at seizure onset of 6 months. Six patients (30%) were male, and 7 were familial cases. We identified pathogenic and likely pathogenic variants in 14 (70%) cases, among them, 6 novel disease gene variants (*KCNQ2*, *CASK*, *WWOX*, *MT-CO3*, *GRIN2D*, and *SLC12A5*). Other genes associated with the disease were *SCN1A* (x2), *SLC2A1*, *ARX*, *CACNA1B*, *PCDH19*, *KCNT1*, and *CHRNA2*. Identification of the genetic causes in 70% of cases confirms the general structure of the etiology of early onset epilepsy and the necessity of using NGS in diagnostics. Moreover, the study describes new genotype-phenotypic correlations in genetic epilepsy. Despite certain limitations of the study, it can be concluded that the genetic etiology of pediatric epilepsy in Kazakhstan is very broad and requires further research.

**Supplementary Information:**

The online version contains supplementary material available at 10.1007/s12035-023-03346-3.

## Introduction

Epilepsy is one of the most common neurological disorders, which affects people of all ages, races, social classes, and geographic locations. Epilepsy is a chronic disease of the brain characterized by a persistent predisposition to the onset of epileptic seizures with neurobiological, cognitive, psychological, and social consequences [1, 2]. In a systematic review and meta-analysis of incidence studies, the pooled incidence of epilepsy was 61.4 per 100,000 person-years, with an overall prevalence of 7.60 per 1000 population; incidence was higher in low/middle-income countries than in high-income countries. Moreover, the incidence of epilepsy is higher in the youngest and oldest age groups, with a rate of 86 per 100,000 per year in the first year of life [3]. Despite a decrease in the prevalence of the disease from 1990 to 2016, epilepsy remains an important cause of disability and death [4].

In the pediatric population, epilepsy is a common neurological disorder affecting approximately 0.5 to 1% of children [5]. According to Jaxybayeva et al. [6] in Kazakhstan, as of October 2020, epilepsy was diagnosed in 15,769 children (aged 0 to 18). Among 450 (32% of total hospitalizations) patients with epilepsy hospitalized in the Department of Pediatric Neurology of the National Scientific Center for Maternal and Child Health of “University Medical Center”; Corporate Found (CF) between 2013 and 2014, 170 of them were diagnosed with epileptic encephalopathy [7]. Early onset epilepsy is associated with poor long-term psychosocial outcomes, and the effects persist into adulthood. People with childhood-onset epilepsy have higher unemployment rates, lower educational attainment, and lower socioeconomic status [5, 8, 9]. According to Lepessova and Myrzaliyeva [10], a high prevalence of epilepsy was observed in regions with unfavorable environmental conditions, which emphasizes the socio-ecological component in the epidemiology of this disease among the pediatric population of Kazakhstan.

Epilepsy is considered a multifactorial disease. The genetic basis of some forms of epilepsy has been put forward for decades and was confirmed by gene mapping and the identification of specific mutations associated with epilepsy syndromes in the 1990s [11–14]. Available data suggests that 70–80% of epilepsy cases have a genetic cause, with the remaining 20–30% associated with acquired conditions such as ischemia, brain injury, tumors, and autoimmune diseases [13, 15]. A review by Wang et al. [16] identified 977 genes associated with epilepsy. At the same time, recent studies have found that genetic causes account for approximately 30% of cases reviewed in the pediatric population [17]. For this reason, genetic testing is widely used today in the practice by epileptologists [18].

Various genetic methods could potentially be applied in the diagnosis of epilepsy. In general, methods such as cytogenetic tests, comparative genomic hybridization (array-CGH), and multiplex ligase-dependent amplification (MLPA) make it possible to diagnose about 10% of pediatric epilepsies and 5% of epileptic encephalopathies [19, 20], since most epilepsies are associated with mutations in individual genes. Currently, traditional Sanger sequencing, which allows direct determination of the nucleotide sequence of a region of a single gene, has been replaced by next-generation sequencing (NGS), which allows simultaneous sequencing of many genes at a relatively low cost [21, 22]. Cohort study conducted in Munich, Germany between 2013 and 2017 indicates that NGS allowed physicians to change the clinical management of 63% of patients with epilepsy [23]. Continuing decline in sequencing costs, the use of WGS is an effective strategy for the clinical diagnosis of early onset epileptic encephalopathy [24]. Another group of researchers from the USA and the UK described two new genes KCNT1 and PIGQ that are pathogenic in Ohtahara syndrome using the WGS method [25].

The first data on whole-genome sequencing in the Kazakh population was published by Akilzhanova et al. [26]. Later, a study conducted among 350 children with early epileptic encephalopathies accompanied by intellectual retardation showed the need for additional funding in healthcare aimed at genetic research of patients with epilepsy; thus, only 15 (4.3%) study participants were able to undergo NGS in the other countries due to financial possibilities, among which 12 (80%) had various genetic mutations and variants associated with the etiology of the disease and resistance to therapy [7].

Thus, this study aims to identify and evaluate genetic variants based on whole-genome sequencing in children with early epilepsy in Kazakhstan.

## Materials and Methods

Study design — retrospective single-center study.

### Ethical Issues

The study was approved by the local commission on bioethics of the “UMC” CF, an extract from Protocol No. 1 dated 06/29/2021.

### Study Participants

The study involved a total of 20 children from unrelated marriages with epilepsy of no known cause and early onset of the disease (according to criteria if the International League Against Epilepsy), hospitalized in the Department of Pediatric Neurology of the National Scientific Center for Maternal and Child Health of the CF “UMC” from July to December 2021, and meeting the following inclusion criteria:

•The onset of a seizures before the age of 3 years;

•The presence of epileptiform discharges on electroencephalography (EEG);

•The absence of structural changes in magnetic resonance imaging of the brain (MRI), which may be the cause of epilepsy;

•Delayed psychomotor development and/or the presence of resistance to antiepileptic drugs and/or the presence of severe epileptic encephalopathy;

•Absence of anomalies found in previous genetic studies (karyotyping);

The criterion for exclusion from the study is the diagnosis in patients before or during the study period of epilepsy with an etiology that could explain the epileptic syndrome. Examples of such etiologies are the brain and meningeal infections, neonatal hypoxic-ischemic encephalopathy, neoplasms, a history of moderate to severe brain injury, and autoimmune diseases affecting the nervous system.

### Whole-Genome Sequencing

Genomic DNA was extracted from whole blood samples using the PromegaTM kit (USA) according to the manufacturer’s protocols. DNA libraries were prepared from 300 ng of genomic DNA using Illumina DNA PCR-Free Library Prep, Tagmentation protocol, with the IDT Indexes for Illumina DNA/RNA UD Set A. DNA libraries were validated using the Qubit ssDNA Assay on the Qubit Fluorometer according to the standard kit protocol. Sequencing was performed on the NovaSeq 6000 high throughput platform using the NovaSeq 6000 S4 Reagent Kit v1.5 (300 cycles) at the National Laboratory Astana.

### Raw Data Preprocessing and Bioinformatics Analysis

Raw data files obtained from the Illumina NovaSeq sequencing platform in binary base call (bcl) format were converted to the fastq file format using the bcl2fastq tool. The quality of the generated sequences has been evaluated using FastQC v.0.11.9 [27] and MultiQC v.1.12 [28]. Sequencing reads were aligned to the human reference genome (NCBI GRCh37, hg19) using Burrows–Wheeler Aligner v.0.7.12 [29]. Picard tools v.2.27.4 used for sorting and marking reads duplicates. Genome Analysis Toolkit (GATK) v.3.8 has been used for genomic variant calling [30]. Genomic variants were annotated using ANNOVAR [31].

### Interpretation of Genetic Variants

Identified genetic variants are described following the nomenclature guidelines of the Human Genome Variation Society (http://www.hgvs.org/mutnomen). Variant interpretation followed the 5-level classification system recommended by the American College of Medical Genetics and Genomics and the Association for Molecular Pathology (ACMG/AMP) and was conducted on the Franklin platform (Franklin by Genoox, Genoox, USA). All possible options identified during the study were evaluated using a three-stage analysis:

1.Variants filtered by their frequency in population control databases;

2.Literature and database review to search for the role of each identified variants in the etiology and course of the disease;

3.Evaluation of the pathogenicity of all identified variants in individual clinical cases.

The following databases are used for variant annotation: OMIM, Human Gene Mutation Database (HGMD), and ClinVar. The pathogenicity of the variants will be predicted using the Polymorphism Phenotyping v2 (PolyPhen-2), MutationTaster, and MutationAssessor prediction algorithms.

## Results

DNAs from total of twenty patients were sequenced on Illumina NovaSeq 6000 platform and the total number of sequenced base pairs yielded from 68,8 to 202,7 Gb with the an average 116 Gb per sample. The mean genome coverage for all samples is 35X. On average, 99.15% of sequencing reads have been properly mapped on a reference genome.

Twenty pediatric patients of the Pediatric Neurology Department of the “University Medical Center” СF with an early onset of epilepsy and an unclear cause of the disease were included in the study. Six (30%) of the patients were male. The age of study participants at the time of recruitment ranged from 4 months to 13 years, with an average age of 34.5 months. Seizure onset ranged from the neonatal period to 3 years of age, with an average of 6 months. Among the probands, a burdened family history of epilepsy was noted in 7 cases.

Of 20 patients recruited in the current study, 7 (35%) presented with focal seizures, 5 (25%) with tonic-clonic seizures, 5 (25%) with generalized seizures, 2 (10%) with polymorphic seizures, and 1 (5%) with myoclonic seizures. Five (25%) patients in the cohort developed therapy-resistant seizures, among them, 3 patients were resistant to valproate, 1 — to phenobarbital, and 1 patient was resistance both to phenobarbital and carbamazepine. Epileptic encephalopathy was diagnosed in 5 patients. Thirteen (65%) patients had a psychomotor delay and 10 were diagnosed with speech delay.

After the whole genome sequencing, pathogenic and likely-pathogenic genetic variants were identified in 14 (70%) out of 20 cases. Clinical, phenotype, and genotype data of 14 patients with genetic etiology of epilepsy are presented in Tables 1 and 2. Clinical and phenotypic data, as well as row data of 6 patients without clinically significant variants are presented in supplementary materials.

**Table 1:** Genetic diagnosis and phenotype in five familial cases

**Case # (gender)**	**Gene (Ref Seq)**	**Genetic variant**	**Inheritance**	**Seizures, type**	**Age of onset, months**	**Phenotype**	**EEG data**	**Brain MRI**	**Additional information**
1 (f)	*KCNQ2* (NM_172107.4)	c.796_797insGTGCTGTTTCTGCCCTGCCCTCCCTGCCTGGGCTGGAACTCAATAA (p.Asp266GlyfsTer80)	AD, heterozygous	Focal, tonic	Infancy	PMD	Diffuse generalized discharge of epileptiform spike-wave activity of a continued nature along the left temporal leads with spread to the right homologous leads, also in the anterior-central regions	Expansion of the lateral ventricles, a cyst of the velum interpositum	Aunt (II-1) and grandmother (I-1) on the proband’s mother’s side, sibling (III-3) with epilepsy
2 (f)	*WWOX* (NM_016373.4)	c.911C>A (p.Ser304Tyr) c.230+1G>T (Splice donor)	AR, compound heterozygous	Focal	1	PMD, spastic ataxia	Spike-slow wave complexes in the right parietotemporal leads	Moderate mixed hydrocephalus, atrophy of the cerebral hemisphere, post-hypoxic encephalopathy	Sibling (II-3) with epilepsy and spastic ataxia, died at the age of 11 months
3 (f)	*CACNA1B* (NM_000718.4)	c.390+1_390+2insACGACACGGAGCCCTATTTCATCGGGATCTTTTGCT (Splice Donor) c.501C>G (p.Asn167Lys)	AR, compound heterozygous	Generalized, tonic	Infancy	PMD, SD	Diffuse delta activity	-	Sibling (III-2) and cousin (III-1) with epilepsy. The proband’s mother (II-4) had seizures during pregnancy. The proband’s grandfather (I-2) had several episodes of seizures after a traumatic brain injury in a car accident
4 (f)	*PCDH19* (NM_001184880.2)	c.1018A>G (p.Asn340Asp)	XL, heterozygous	Clonic-tonic	11	PMD, SD, aggression, resistance to valproate	Spike-slow wave complexes in the central-frontal-temporal region bilaterally	-	Three female siblings (II-2-4) with epilepsy
5 (f)	*SLC12A5* (NM_020708.5)	c.2930A>G (p.Gln977Arg)	AD, heterozygous	Generalized	36	PMD, SD	Periodic diffuse slow waves, low-amplitude single and grouped spike-slow wave complexes in the right central and temporal leads, rarely spreading to the right parts of the brain	Minimal post-hypoxic changes in the white matter of the cerebral hemispheres	Two female siblings (III-1,2) with epilepsy, one of them died at the age of 3 y 4 m (III-2). Paternal cousins (III-4,5) died in infancy from cerebral palsy

Note: *AD*, autosomal dominant; *AR*, autosomal recessive; *XL*, X-linked; *PMD*, psychomotor delay; *SD*, speech delay; *f*, female

**Table 2:** Genetic diagnosis and phenotype in 9 non-familial cases

**Case # (gender)**	**Gene (Ref Seq)**	**Genetic variant**	**Inheritance**	**Seizures, type**	**Age of onset, months**	**Phenotype**	**EEG data**	**Brain MRI**
6 (m)	*CASK* (NM_001367721.1)	c.2301T>G c.2300T>G (p.Phe767Gly)	XLD/XLR, hemizygous	Generalized, tonic	6	PMD, EE	Diffuse, bilateral-synchronous and asynchronous peak/spike-slow wave complexes	Diffuse changes in the white matter of the cerebral hemisphere (post-hypoxic genesis), mixed hydrocephalus, retrocerebellar cyst, cyst of the right choroidal fissure
7 (f)	*SLC2A1* (NM_006516.4)	c.988C>T (p.Arg330*)	AD, heterozygous	Generalized, clonic-tonic	3	PMD, SD, EE, microcephaly	Central leads, spike-slow wave complexes	Diffuse changes in the white matter of the cerebral hemisphere
8 (m)	*ARX* (NM_139058.3)	c.1612A>G (p.Lys538Glu)	XLR, hemizygous	Polymorphic	6	PMD, SD, microcephaly, hypotony	Low-amplitude hypsarrhythmia — constant multifocal, asynchronous, epileptiform activity in the form of spike-slow wave complexes with a focus in the frontal, separately in the temporal and central leads	Post-hypoxic changes in the cerebral hemispheres, mixed hydrocephalus
9 (f)	*MT-CO3*	c.172G>A (p.Trp58*)	Mi, homoplasmy	Generalized, tonic	1	PMD, SD, acidosis, excess subcutaneous fat	Grouped complexes of a spike-slow wave in the frontal leads with spread to the middle-temporal leads bilaterally	Symmetrical focal changes in the structure of the thalamus of a metabolic/neurodegenerative nature (Leigh syndrome), moderate combined hydrocephalus, retrocerebellar cyst
10 (f)	*GRIN2D* (NM_000836.4)	c.3550G>A (p.Ala1184Thr)	AD, heterozygous	Polimorphic	7.5	SD	Lateralized to the right leads diffuse rhythmic theta-like wave with the beginning in the parietal and temporal leads gradually turning into low-frequency delta waves and spreading to the left parts of the brain	Cysts of the septum pellucidum
11 (f)	*KCNT1* (NM_020822.3)	c.1421G>A (p.Arg474His)	AD, heterozygous	Clonic-tonic	1	PMD, SD, resistance to fenobarbital, carbamazepine	Rhythmic beta/alpha-like waves in the right occipital and separately in some seizures in the right temporal leads, with diffuse propagation, gradually turning into theta waves	Post-hypoxic changes in the white matter of the cerebral hemispheres, moderate external hydrocephalus
12 (m)	*CHRNA2* (NM_000742.4)	c.612G>A (p.Trp204*)	AD, heterozygous	Focal, clonic	Infancy	SD, status epilepticus, resistance to fenobarbital	Bursts of delta waves with a maximum amplitude in the central and temporal leads	Subatrophy of the cerebral hemispheres, bilateral convexital subdural hygroma more pronounced on the right
13 (f)	*SCN1A* (NM_001165963.4)	c.3706-2A>C (Splice acceptor)	AD, heterozygous	Focal, clonic	7	PMD, SD	Continued deceleration in the right frontal leads	Intracranial hypertension
14 (m)	*SCN1A* (NM_001165963.4)	c.3982T>C (p.Ser1328Pro)	AD, heterozygous	Focal, clonic	4	Resistance to levetiracetam	An increase in the index of diffuse slow delta waves, single sharp waves were also recorded in the occipital, separately in the frontal leads	-

Note: *AD*, autosomal dominant; *XLD*, X-linked dominant; *XL*, *XLR*, X-linked recessive; *Mi*, mitochondrial; *PMD*, psychomotor delay; *SD*, speech delay; *EE*, epileptic encephalopathy; *m*, male; *f*, female

**Fig. 1:**
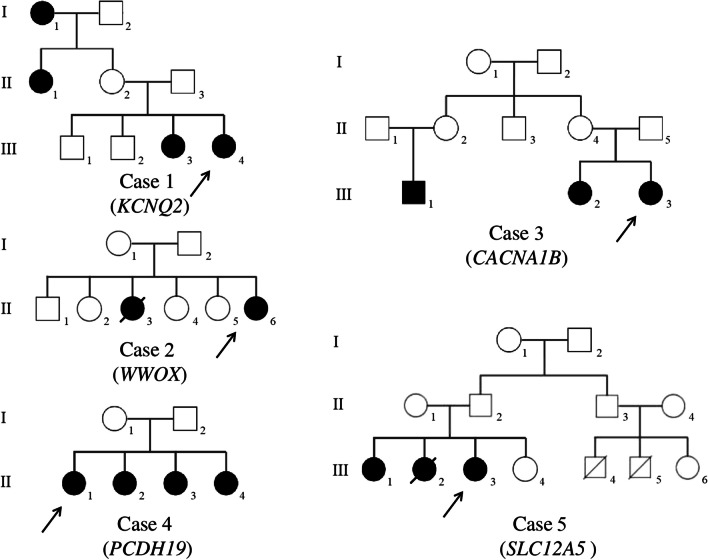
Pedigree charts of the cases with family epilepsy history (created with BioRender.com)

Among 14 patients with identified pathogenic/likely pathogenic genetic variants, a family history of epilepsy was in 5 cases (cases # 1 to 5). Clinical, phenotype, and genotype data of five cases with family history of epilepsy are presented in Table 1; pedigree charts of these cases are presented in Fig. 1.

Previously undescribed variants were found in cases 1, 2, 5, 6, 9, and 10 (*KCNQ2*, *WWOX*, *SLC12A5*, *CASK*, *MT-CO3*, and *GRIN2D* genes, respectively). Case 1 is a girl, aged 2 years at the time of genetic analysis. For the first time, convulsions were registered on the second day of life. In this patient, the study revealed a pathogenic variant in the *KCNQ2* gene. This variant was a 46 bp insertion followed by a frameshift in the exon 5.

Case 2 is a girl who had her first seizures at the age of 1 month. During counseling, complaints were of seizures up to 30 times a day for 30 s. This patient was found to have compound heterozygous variants in the *WWOX* gene. Both genetic variants have not previously been described.

Case 5 is a girl with onset of seizures at 3 years old; however, epileptic activity on the EEG was noted earlier. The genetic variant in the proband was represented by a missense mutation in the *SLC12A5* gene in the heterozygous state.

Case 6 is a boy with an onset of seizures at 6 months. Two nucleotide substitutions (missense mutations) were found in the *CASK* gene, leading to a change in the amino acid sequence at position Phe767Gly.

Case 9 is a girl with no family history of epilepsy and a previously undescribed variant in the mitochondrial gene, the cytochrome C oxidase 3 (*MT-CO3*) gene. This girl also had excess subcutaneous adipose tissue, opticopathy, and partial atrophy of the optic disc. Seizures in this patient were managed by the admission of valproate.

Case 10 is a girl whose first seizure was at 7.5 months on the background of elevated temperature. This patient had previously been screened for microdeletion/microduplication syndrome by MLPA which was negative. In this girl, a previously undescribed and pathogenic variant was identified in the *GRIN2D* gene.

Resistance to at least one of the antiepileptic drugs was found in cases 4, 11, 12, and 14. Case 4 had resistance to valproate, but seizures were partially controlled by oxcarbazepine. Case 11, with a pathogenic variant in the *KCNT1* gene, was resistant to phenobarbital and carbamazepine, while a partial positive response was achieved with topiramate and vigabatrin. Case 12 was resistant to phenobarbital as well as to low doses of valproate, while seizure relief was achieved by administration of 150 mg/day of valproate in combination with levetiracetam at a dosage of 100 mg/day. Case 14 initially received levetiracetam, but the frequency of seizures was taken into account until the onset of status epilepticus. This patient is currently receiving a combination of valproate and carbamazepine.

## Discussion

The study describes the results of the first whole genome sequencing among children with early onset epilepsy in Kazakhstan. Pathogenic and likely pathogenic genetic variants were identified in 70% of cases during the study (14/20). This indicator was higher than in previous studies. So, in one study, researchers found the presence of clinically significant genetic mutations in 20% of patients (among 118 children with epilepsy) in Sweden [32]. In other studies from South Korea and Scotland, with more detailed inclusion criteria, NGS detected genetic abnormalities in 37.8% and 24% of patients with early-onset epilepsy, respectively [33, 34]. The higher percentage of clinically relevant genetic variants identified in this study may be explained by the strict inclusion criteria and the small sample size.

The following genes have been identified as epilepsy etiology: *ARX*, *CACNA1B*, *CASK*, *CHRNA2*, *GRIN2D*, *KCNQ2*, *KCNT1*, *MT-CO3*, *PCDH19*, *SCN1A* (x2), *SLC2A1*, *SLC12A5*, and *WWOX*. In an early study, Jaxybayeva et al. [6] identified a genetic cause in 80% (12/15) of children with early seizures using whole-exome sequencing in Kazakhstan (*CDKL5* (x3), *SCN1A* (x2), *MECP* (x2), *STXBP1*, *UBE3A*, *PCDH19*, *FOLR1*, *PNPO*). Summing up the results from the present and the above mentioned studies, which is acceptable due to relatively similar inclusion criteria, the overall detection rate for the genetic etiology of early-onset epilepsy was 74.3% (26/35). And most often clinically significant genetic variants have been identified in *CDKL5* (x3), *SCN1A* (x3), *MECP* (x2), and *PCDH19* (x2) genes.

*KCNQ2-*related neonatal-onset developmental and epileptic encephalopathy is characterized by mostly tonic seizures beginning in the first week of life [35], which coincided with the clinical presentation of case 1. GLUT1 deficiency syndrome caused by mutations of the *SLC2A1* gene is characterized by early infantile epilepsy, developmental delay, microcephaly, complex movement disorders, and various paroxysmal neurological phenomena [36, 37], which also corresponds to the indicated case 7. This study also expands the understanding of the clinical features of mutations in the *ARX* gene. X-linked infantile spasms syndrome, West syndrome, Ohtahara syndrome, and myoclonic epilepsy syndromes may be associated with *ARX* gene mutations and characterized by pediatric epilepsy, intellectual disability, developmental and speech delay, intractable seizures, hypotonia, psychiatric abnormalities, brain malformations, and ambiguous genitalia [38–41]. Previously described variants associated with *MT-CO3* (COX) gene were represented by the following clinical characteristics: MELAS syndrome, rhabdomyolysis, and mitochondrial myopathy with lactic acidosis and one with Leigh syndrome ([42–46]. In this study, we expand the clinical manifestation of the *MT-CO3*-related Leigh syndrome. Moreover, current research confirms and expands the genotype-phenotypic correlation of the following conditions: *PCDH19*-related epilepsy [47], *GRIN2D*-related developmental and epileptic encephalopathy [48], *KCNT1*-related epilepsy [49], *CHRNA2*-related autosomal dominant sleep-related hypermotor epilepsy [50], *SCN1A*-related epilepsy [51], *SLC12A5*-related epilepsy [52].

At the same time, case 6 differed from the previously described phenotypic manifestations. So, variants in the *CASK* gene were mainly the cause of epilepsy with microcephaly, pontine, and cerebellar hypoplasia, and typically affect females [53]. The previously described genotype-phenotypic correlation of neurological diseases associated with variants in the *WWOX* gene was associated with spinocerebellar ataxia type 12, early infantile epileptic encephalopathy, and autism spectrum disorder [54, 55]. In current study, *WWOX*-related epilepsy was characterized by psychomotor retardation, spastic ataxia, and encephalopathy of the cerebral hemispheres on the MRI picture. Individuals affected by *CACNA1B* variants presented with epileptic encephalopathy, severe neurodevelopmental delay, hyperkinetic movement disorder (myoclonus-dystonia syndrome), postnatal microcephaly, and hypotony [56, 57]. However, case 6 with compound heterozygous variants in *CACNA1B* had generalized tonic seizures, psychomotor, and speech delay.

One of the important results of identifying the etiology of epilepsy is the application of precision medicine based on genetic testing results. Among the identified 14 cases of genetic epilepsy, targeted therapy was indicated in 8 (57%) cases [22, 58]. The results obtained confirm the importance of using genetic diagnostic methods, especially NGS, for the management of patients with epilepsy.

Undoubtedly, the study was not population-based, had a limited number of participants (*n*=20), and had strict inclusion criteria. In this regard, the obtained high percentage of detection of genetic etiology does not reflect the genetic structure of epilepsy in Kazakhstan. However, the inclusion criteria adapted during the study makes it possible to determine a cost-effective algorithm for diagnosing epilepsy.

## Conclusion

The data obtained indicate the high significance of NGS in the diagnosis and management of patients with early-onset epilepsy. Moreover, the study complements the existing knowledge about the genotype-phenotype correlation in epilepsy and highlights the need for the introduction and expansion of NGS diagnostics in Kazakhstan.

## Supplementary Information

ESM 1

## Data Availability

All data available by request to corresponding author. Sequencing data for individual samples have been deposited at National Center for Biotechnology Information Sequence Read Archive under accession number PRJNA954058 (https://www.ncbi.nlm.nih.gov/bioproject/PRJNA954058).
