# Correction: Whole-Genome Sequencing Among Kazakhstani Children with Early-Onset Epilepsy Revealed New Gene Variants and Phenotypic Variability

**DOI:** 10.1007/s12035-024-04544-3

**Published:** 2024-10-12

**Authors:** Mirgul Bayanova, Aidos K. Bolatov, Assiya Bazenova, Lyazzat Nazarova, Alissa Nauryzbayeva, Naanlep Matthew Tanko, Saule Rakhimova, Nazerke Satvaldina, Diana Samatkyzy, Ulan Kozhamkulov, Ulykbek Kairov, Ainur Akilzhanova, Dos Sarbassov

**Affiliations:** 1 University Medical Center CF, Kerey-Zhanibek Handar St. 5/1, Z05P3Y4 Astana, Kazakhstan; 2 Astana Medical University, Beybitshilik St. 49A, Z10K9D9 Astana, Kazakhstan; 3 Department of Biomedical Sciences, School of Medicine, Nazarbayev University, Astana, Kazakhstan 010000; 4 Center for Life Sciences, National Laboratory Astana, Nazarbayev University, Kabanbay batyr Ave 53, Astana, Kazakhstan 010000; 5 School of Sciences and Humanities, Nazarbayev University, Kabanbay batyr Ave 53, Astana, Kazakhstan 010000

**Correction: Molecular Neurobiology (2023) 60:4324-4335**

**https://doi.org/10.1007/s12035-023-03346-3**

In the original version of this article, the family name of Diana Samatkyzy were incorrectly spelled as “Samakyzy”. The correct surname should be written as “Samatkyzy”.

The original article has been corrected.

