# Supplementary material for: Whole-Genome Sequencing Among Kazakhstani Children with Early-Onset Epilepsy Revealed New Gene Variants and Phenotypic Variability
Source: Mol Neurobiol. 2023 Apr 24;60(8):4324–35. doi: 10.1007/s12035-023-03346-3 (PMC10293429; doi:10.1007/s12035-023-03346-3)
Supplement: Supplementary file 1 — ESM 1 [file 12035_2023_3346_MOESM1_ESM.docx]

**Table 1. Phenotype of 6 cases without confirmed genetic diagnosis**

| **Case # (gender)** | **Seizures, type** | **Age of onset, months** | **Phenotype** | **EEG data** | **Brain MRI** | **Additional information** |
| --- | --- | --- | --- | --- | --- | --- |
| 25UMC (m) | focal | 9 | PMD | low-amplitude acute-slow wave complexes recorded according to the type of benign epileptiform patterns of childhood in the central leads bilaterally. | minimal posthypoxic changes in the white matter of the cerebral hemispheres, moderate atrophic changes in the poles of the temporal lobes. | resistance to valproic acid |
| 30UMC (f) | focal | Infancy | PMD | regional pathological and epileptiform activity was not registered during the study. | moderate asymmetry of the hippocampus, diffuse changes in the structure of the head of the right hippocampus (probably postictal changes). |  |
| 31UMC (m) | focal | 6 | PMD, SD, EE | In wakefulness, seizures were registered in the form of staring, chewing, chaotic hand movements. The EEG at that time recorded alpha-like waves in the occipital leads | posthypoxic encephalopathy, a single neuroglial cyst in the occipital lobe on the left. |  |
| 33UMC (f) | focal | 9 | SD, EE | As sleep deepens, the index of slow waves in the delta range increases, and the reduction of sleep spindles occurs (stage 3).  Against this background, a constant continued regional bilateral-synchronous, epileptiform activity is recorded, in the form of high-amplitude slow irregular waves and acute-slow wave complexes in the central parietal and temporal leads (fuzzy intermittent reversion P3P4, T5, T6) with an amplitude of up to 500-600 microvolts. | diffuse changes in the white matter of the cerebral hemispheres, posthypoxic genesis. | intolerance to valproic acid |
| 37UMC (f) | focal | 10 | - | During sleep, periodic pathological activity is recorded in the form of acute-slow wave complexes in the fronto-central leads, with an amplitude of 300-400 μV, bilaterally synchronously as part of physiological sleep transits, and periodic diffuse slowing of the delta range is also recorded, with an amplitude of up to 500 μV, with a topical maximum in fronto-central leads. | mixed hydrocephalus with a predominance of internal, cyst of the intermediate sail | resistance to valproic acid |
| 55UMC (f) | Generalized | 2 | - | epileptiform activity in the form of complexes of sharp waves in the fronto-central leads on the right. | external hydrocephalus | male sibling with epilepsy, congenital malformations of the CNS pachygyria, |

Link to the row data of WGS:
